# *In vivo* biodistribution and toxicity of intravesical administration of quantum dots for optical molecular imaging of bladder cancer

**DOI:** 10.1038/s41598-017-08591-w

**Published:** 2017-08-24

**Authors:** Ying Pan, Timothy Chang, Gautier Marcq, Changhao Liu, Bernhard Kiss, Robert Rouse, Kathleen E. Mach, Zhen Cheng, Joseph C. Liao

**Affiliations:** 1 Department of Urology, Stanford University School of Medicine, Stanford, CA 94305 USA; 2 Veterans Affairs Palo Alto Health Care System, Palo Alto, CA 94304 USA; 3 Department of Radiology and Molecular Imaging Program at Stanford, Stanford University School of Medicine, Stanford, CA 94305 USA; 4 Department of Pathology, Stanford University School of Medicine, Stanford, CA 94305 USA

## Abstract

Optical molecular imaging holds the potential to improve cancer diagnosis. Fluorescent nanoparticles such as quantum dots (QD) offer superior optical characteristics compared to organic dyes, but their *in vivo* application is limited by potential toxicity from systemic administration. Topical administration provides an attractive route for targeted nanoparticles with the possibility of minimizing exposure and reduced dose. Previously, we demonstrated successful *ex vivo* endoscopic imaging of human bladder cancer by topical (i.e. intravesical) administration of QD-conjugated anti-CD47. Herein we investigate *in vivo* biodistribution and toxicity of intravesically instilled free QD and anti-CD47-QD in mice. *In vivo* biodistribution of anti-CD47-QD was assessed with inductively coupled plasma mass spectrometry. Local and systemic toxicity was assessed using blood tests, organ weights, and histology. On average, there was no significant accumulation of QD outside of the bladder, although in some mice we detected extravesical biodistribution of QD suggesting a route for systemic exposure under some conditions. There were no indications of acute toxicity up to 7 days after instillation. Intravesical administration of targeted nanoparticles can reduce systemic exposure, but for clinical use, nanoparticles with established biosafety profiles should be used to decrease long-term toxicity in cases where systemic exposure occurs.

## Introduction

Optical molecular imaging has emerged as a powerful tool to improve endoscopic detection of cancer. Molecular imaging may significantly improve cancer detection in the gastrointestinal and urinary tracts where identification of suspicious lesions currently relies mainly on white light endoscopy, which has well-known shortcomings including suboptimal detection of flat lesions and inadequate delineation of tumor boundaries that may impair complete tumor resection, thereby contributing to cancer recurrence and progression^1–5^. A combination of cancer-specific imaging agents and optical imaging technologies can augment cancer detection and resection^6–8^. For successful clinical translation, molecular imaging agents need to be safe, cancer-specific, and coupled with a clinically approved imaging device.

One imaging strategy is to use fluorescently labeled monoclonal antibodies to target surface proteins on cancer cells. Several ongoing clinical trials are assessing the use of targeted organic fluorophores such as IR-800CW, Alexa Fluor 488 and Cy5 to improve cancer imaging^8, 9^. However, as an alternative to organic dyes, imaging tags based on nanoparticles have attracted wide interests given their superior optical properties^6, 10–12^. Quantum dots (QD) are a family of nanoparticles made of semi-conductive core materials such as cadmium (Cd) and selenium (Se) coated with a zinc sulfide (ZnS) shell^13^. Compared to organic dyes, QD are ~20-fold brighter and 100-fold more resistant to photobleaching^14^. Unlike organic fluorophores, which have narrow excitation and emission peaks (<40 nm), QD have broad excitation spectra and narrow emission peaks. The fluorescence emission spectrum, ranging from ultraviolet to near-infrared, depends principally on the particle size^15, 16^. However, the optical properties can also be tuned without changing the particle size through manipulations of the composition and internal structure^17^. As such QD can be synthesized to be compatible with existing clinical systems for detection of fluorescence. Clinical translation of QD has been limited by concerns of tissue persistence and potential long-term toxicity due to the heavy metal core. While QD are often encased in polymer shells to reduce toxicity^18, 19^, long-term QD persistence could lead to degradation of the shell and exposure of the Cd/Se core that can generate reactive oxygen species with resulting cellular and subcellular toxicity^20–22^. Recent studies of intravenous administration of QD in mice^23^, rats^24^ and non-human primates^25^ have demonstrated persistence of QD for up to 90 days in liver, kidneys, and spleen but did not find significant hematological or organ toxicity.

Optical molecular imaging could significantly enhance detection and resection of bladder tumors. Bladder cancer is the 5^th^ most common cancer^26^ and is primarily managed cystoscopically for early stage (i.e. Ta, T1, TIS) non-muscle invasive disease^27^. Most bladder tumors are identified by white light cystoscopy. Blue light cystoscopy with imaging agent hexaminolevulinate (HAL), a precursor of protoporphyrin IX (PPIX), is approved for use to improve bladder cancer detection^28^. Intravesically instilled HAL is taken up by the bladder epithelium where PPIX accumulates preferentially in tumor cells and fluoresces red under blue light. Blue light cystoscopy with HAL has improved the sensitivity of bladder cancer detection, however it has several shortcomings notably suboptimal specificity in the context of inflammation^29, 30^.

The urinary bladder is easily accessible and highly amenable to topical (i.e. intravesical) drug administration. Advantages of topical drug administration include direct instillation of therapeutic and diagnostic agents to the organ of interest at high concentration while minimizing systemic toxicity. For treatment of bladder cancer, live bacillus Calmette–Guérin and mitomycin are established intravesical immunotherapy and chemotherapy agents, respectively, to reduce the recurrence of localized bladder cancer after transurethral resection^27^.

CD47 is a surface protein widely expressed in the majority of cancers including bladder^31^. Differential expression of CD47 between cancer and the normal urothelium in the bladder makes it a good cancer imaging target. We previously reported targeted molecular imaging of human bladder cancer with QD-conjugated antibody against CD47 (anti-CD47-QD) in fresh intact human bladders from radical cystectomy for invasive bladder cancer^10^. To mimic the potential clinical use, anti-CD47-QD was instilled intravesically in cystectomy specimens followed by optical imaging with a clinical blue light cystoscope. Anti-CD47 antibodies were found to be confined to the surface of bladder tumors and absent on normal urothelium, suggesting little if any anti-CD47 tissue penetration in *ex vivo* tissue^10^. The combination of anti-CD47-QD and blue light cystoscopy demonstrated promising diagnostic accuracy for bladder cancer in the *ex vivo* validation study.

Direct bladder instillation and removal of an imaging agent could minimize systemic uptake and subsequent toxicity. This route of direct topical application may allow for clinical translation of imaging agents such as anti-CD47-QD. In this study we assessed the *in vivo* biodistribution and toxicity of intravesically instilled QD and anti-CD47-QD in mice. A better understanding of biodistribution of intravesically instilled agent is key to the potential translation of anti-CD47-QD and is relevant to other targeted nanoparticles.

## Results

### Biodistribution of intravesical anti-CD47-QD by ICP-MS

**Figure 1:**
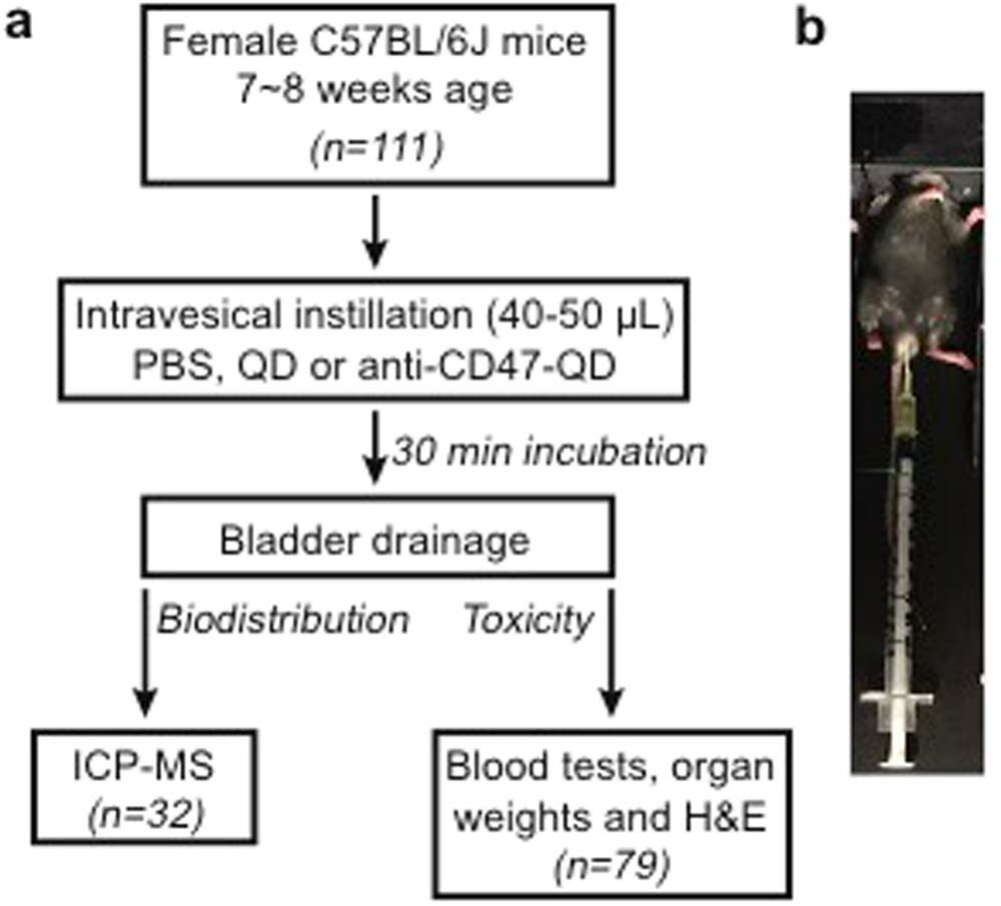
Experimental design for *in vivo* biodistribution and toxicity studies of intravesical imaging agents. (**a**) Schematic of quantum dot (QD) and anti-CD47-QD instillation in mice. (**b**) Photograph of mouse during incubation after transurethral instillation through catheter with syringe attached to act as air block to prevent efflux. ICP-MS, inductively coupled plasma mass spectrometry.

Figure 1 shows the overall experimental design for the *in vivo* biodistribution and toxicity studies in the mouse model (n = 29). As Cd is found in the core of QDs, the biodistribution of anti-CD47-QD was determined by measuring differences in Cd levels between treated and untreated mice using inductively coupled plasma mass spectrometry (ICP-MS). Cd levels in blood, heart, lung, spleen, kidneys, liver and bladder were quantitatively measured. Endogenous Cd levels in blood and organs of untreated mice (n = 3) were measured as baseline control. The biodistribution experiment was conducted sequentially in two cohorts with same protocol. An additional 0-hour time point post-instillation was added in Cohort 2. In Cohort 1 (n = 9), three mice were sacrificed and blood and organs harvested at each time point post instillation (1-, 4- and 24-hours). Cohort 2 (n = 20) included an additional time point immediately post instillation (0-hour), and five mice were sacrificed at each time point (0-, 1-, 4- and 24-hours). The total amount of Cd detected in blood and organs of each mouse is shown in Supplementary Table S1.

**Figure 2:**
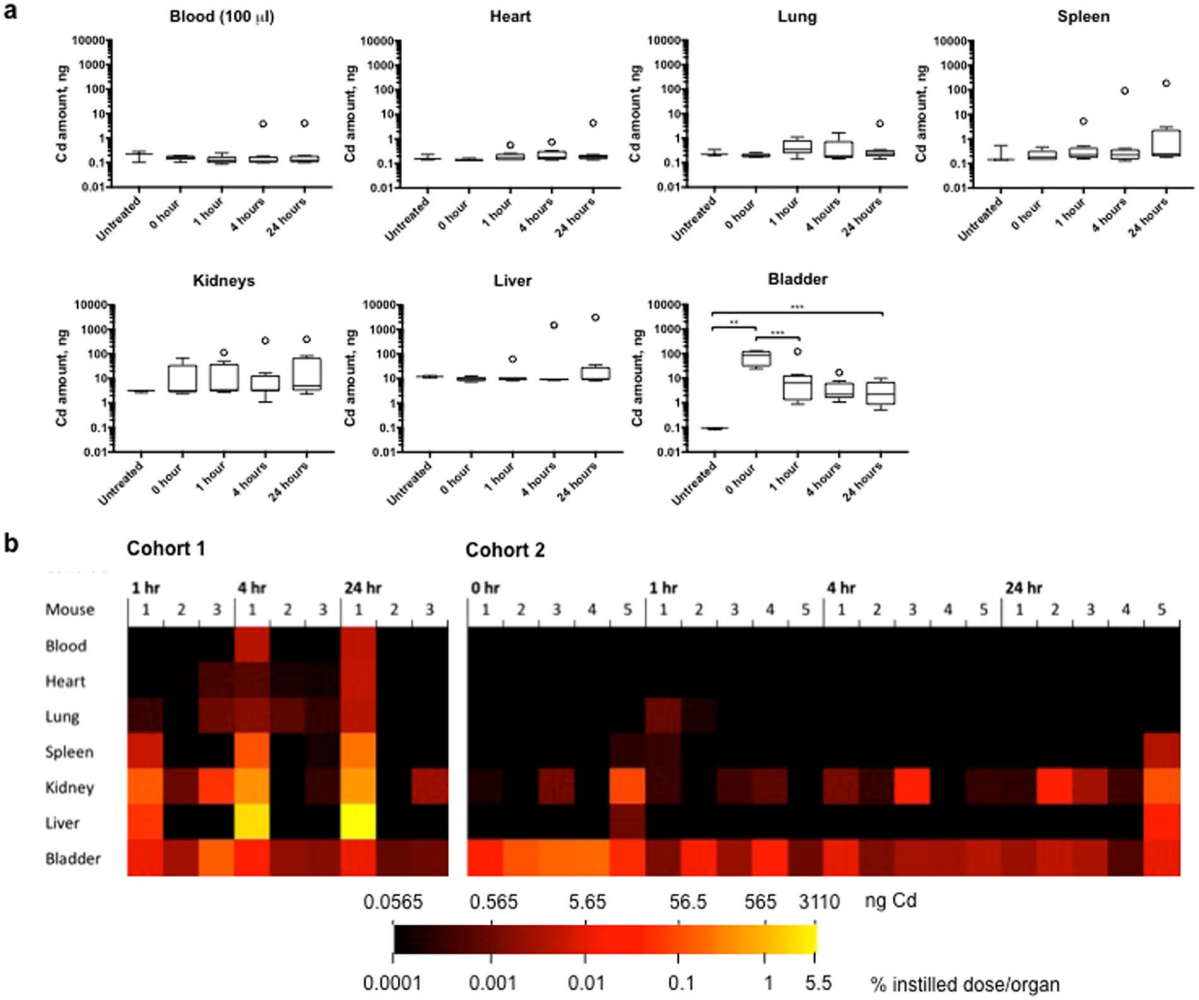
Cadmium (Cd) in blood and organs of untreated and anti-CD47-QD instilled mice after intravesical exposure. (**a**) Box and whisker plots of Cd amount (ng) in blood (100 µl) and whole organs collected from untreated, and anti-CD47-QD-instilled mice at 0-, 1-, 4- and 24-hour post instillation. **Denotes statistically significant difference. (**b**) Heat map demonstrating Cd distribution in each mouse in anti-CD47-instilled mice for Cohorts 1 and 2. Heat map data are displayed on a logarithmic scale with baseline Cd level subtracted. Each block represents amount of Cd in blood (100 µl) or organs in ng and % instilled dose/organ.

Overall, the bladder was the only organ with a statistically significant increase in Cd compared to untreated animals (Fig. 2a). After an initial spike of Cd immediately after instillation (0-hour group), the Cd level in the bladder decreased significantly (*p* = 0.0067) after 1-hour and remained unchanged through 24-hours post instillation (1-hour vs 4-hours, p = 0.81; 4-hours vs 24-hours, p = 0.99). At 24-hour post instillation the Cd level was still significantly elevated compared to the untreated group (p = 0.0027). For the remaining organs, there were no statistically significant increases in Cd concentration among untreated, 0-hour, 4-hour, and 24-hour groups (blood, p = 0.63; heart, p = 0.10; lung, p = 0.48; spleen, p = 0.51; kidneys, p = 0.73; and liver, p = 0.42).

While the average levels of Cd in organs outside of the bladder did not meet the statistical threshold for significant elevation, notable levels of Cd were found in some mice. Figure 2b shows a heatmap of Cd biodistribution for each organ. Mouse 1 in the 24-hour group of Cohort 1 had the highest Cd level, retaining 6.6% on the initial instilled dose, 83.8% of which was in the liver (5.5% total instilled dose). In Cohort 1, four out of the nine mice had systemic Cd accumulation greater than 0.30% of the instilled dose (0.30% to 6.56% of instilled dose). The remaining 5 mice had total Cd accumulations of less than 0.006% of the instilled dose. In Cohort 2, no mouse accumulated more than 0.05% of the instilled dose of Cd in any of the measured organs outside of the urinary tract (bladder and kidneys). The largest accumulation outside of the urinary tract of Cd was 0.048% in the liver of Mouse 5 in the 24-hour group, and the second highest accumulation outside of the urinary tract was about 0.0056% instilled dose in the spleen of the same mouse.

### Toxicity of intravesical QD and anti-CD47-QD

**Figure 3:**
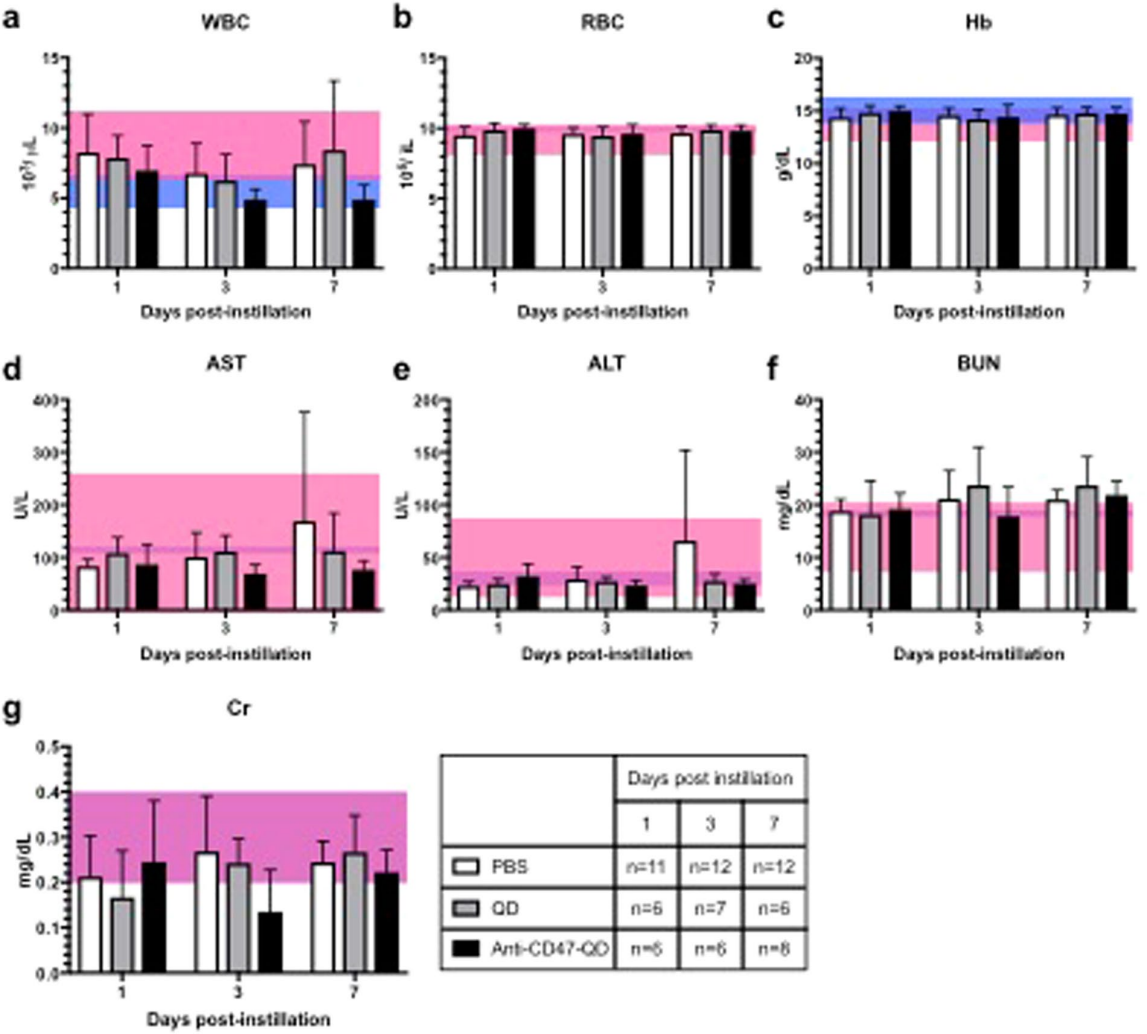
Blood test results from mice sacrificed post intravesical exposure to PBS, QD, and anti-CD47-QD. The pink region represents the normal range for mice as provided by the vendor, and the blue region represents the range (±1 standard deviation from the mean) we obtained in this study for our 3 untreated mice. Areas of overlap between the two are purple. Error bars represent one standard deviation above the mean. (**a**) WBC, white blood cells. (**b**) RBC, red blood cells. (**c**) Hb, hemoglobin. (**d**) AST, aspartate aminotransferase. (**e**) ALT, alanine aminotransferase. (**f**) BUN, blood urea nitrogen. (**g**) Cr, creatinine. The results show no changes in blood count, liver enzymes and kidney function at 1-, 3- and 7-days after QD or anti-CD47-QD instillation. The table indicates number of mice analyzed at each time point.

**Figure 4:**
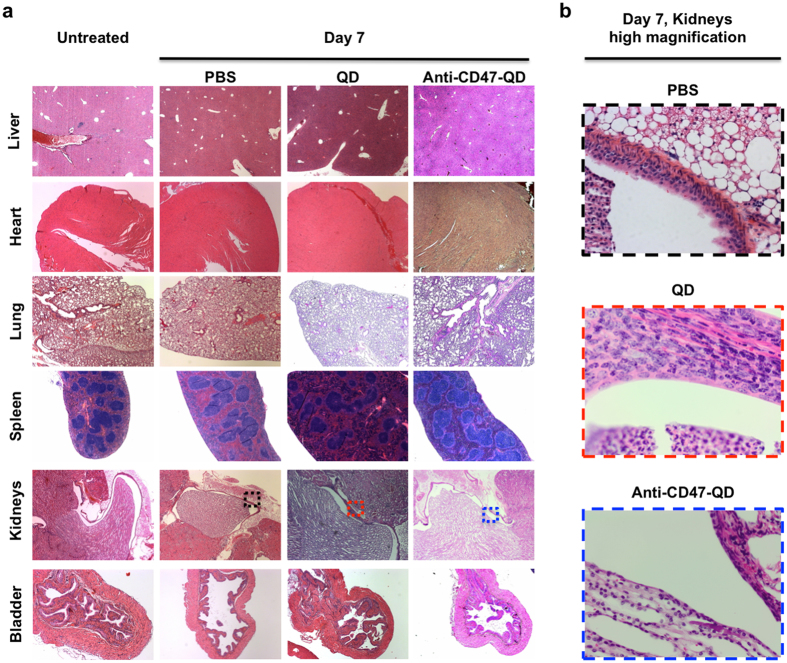
Histological images of liver, heart, lung, spleen, kidneys and bladder from untreated, PBS, QD and anti-CD47-QD exposed mice. No abnormalities were observed in liver, heart, lung spleen or bladder at any time point. Inflammation was found only in kidneys of QD-instilled mice. (**a**) Representative low magnification H&E images of organs collected from untreated mice and PBS-, QD- and anti-CD47-QD-instilled mice at 7-day post instillation. (**b**) High magnification images of renal pelvis of PBS- (black box), QD- (red box) and anti-CD47-QD-treated (blue box) mice. The image of QD-treated mouse (red box) shows increased inflammatory cells in the renal pelvic lumen.

We investigated the potential toxicity associated with intravesical exposure to unconjugated QD and the molecular imaging agent anti-CD47-QD. A total of 79 mice were examined, including intravesical instillation with unconjugated QD (n = 19), anti-CD47-QD (n = 20), PBS only (n = 35), and untreated controls (n = 3). Mice were sacrificed at 1, 3 or 7 days post instillation and blood, heart, liver, spleen, lung, kidneys and bladder harvested. Blood was analyzed for white blood cell (WBC) count, red blood cell (RBC) count, hemoglobin (Hb), blood urea nitrogen (BUN), creatinine (Cr), aspartate aminotransferase (AST), and alanine aminotransferase (ALT) (Fig. 3). Organs were weighed (Supplementary Table S2) and processed for hematoxylin and eosin (H&E) analysis to assess histological differences between the treated and untreated groups (Fig. 4).

No statistically significant differences were observed at any time point in the blood test results of the control (PBS) and treated groups (QD and anti-CD47-QD). ANOVA analysis of blood tests indicated no significant difference in WBC (p = 0.06), RBC (p = 0.46), Hb (p = 0.63), BUN (p = 0.08), or Cr (p = 0.19) between control and treated groups at day 1, day 3 and day 7. Nonparametric Kruskall-Wallis test was performed for the liver enzymes and showed no differences between groups: AST (p = 0.85) and ALT (p = 0.64) (Fig. 3).

There were no significant changes in organ weight of livers, kidneys, lungs, hearts, and bladders (Supplementary Table S2) between mice for each group (p = 0.19, p = 0.74, p = 0.93, p = 0.68, and p = 0.30, respectively). Spleen weight was elevated in the day 3 QD group (96 mg [interquartile range 83–112]) compared to the day 3 anti-CD47-QD group (72 mg [interquartile range 65–76], p = 0.007). However, this elevation was not significantly different from the PBS control group.

The histology of the organs collected from QD- and anti-CD47-QD instilled mice at all time points was normal, with the exception for the presence of inflammatory cells in the renal pelvis (submucosal and luminal) of 5 of the 19 QD-instilled mice (Fig. 4). Inflammatory cells in the renal pelvis were not noted in the mice instilled with anti-CD47-QD. The mice with renal inflammation included 1 out of 6 mice from the 1-day post instillation group, 3 out of 7 mice from the 3-day group, and 1 out of 6 mice from the 7-day group. Despite the inflammation noted in the kidneys, the bladders of all mice exposed to QD- or anti-CD47-QD appeared normal with intact mucosa.

## Discussion

The concern for toxicity has been a translational barrier for nanoparticle-based imaging agents. For QD, potential toxicity can depend on a combination of factors including particle composition, dose, surface chemistry and route of administration. We previously evaluated intravesical instillation of a QD-labeled molecular imaging agent in *ex vivo* human bladders^10^. We found QD-labeled anti-CD47 specifically localize to the luminal surface of bladder cancer and is a promising imaging agent. To examine whether intravesical administration is a safe route for potential clinical use of QD-labeled antibodies, we examined the *in vivo* biodistribution and toxicity of intravesical QD and anti-CD47-QD in mice. Overall, we did not find statistically significant evidence of systemic anti-CD47-QD or toxicity after intravesical exposure. Nevertheless, in some mice considerable levels of QD were found outside of the bladder.

Several studies have investigated the biodistribution and toxicity of intravenous (IV) injected QD. In mice^32, 33^ and rhesus macaque^25^, QD accumulation and persistence was found in liver, kidney and spleen after IV administration. Yet, consistent with this study, acute toxicity was not detected despite the presence of QD. For target organs such as lung, skin, GI tract^34^, and bladder, molecular agents can be administered topically to minimize systemic exposure For example, Roberts *et al*. assessed pulmonary toxicity and biodistribution of unconjugated QD following intratracheal instillation in rat lung. QD was not removed after instillation to allow for long-term exposure. Detection of QD in lung and associated lymph nodes as well as kidney accumulation up to 28 days post instillation were noted. While dose dependent lung injury and inflammation was found, there was no evidence of QD in blood, liver, spleen or brain^35^. Gopee *et al*. investigated skin penetration of QD, and found a low but significant level of Cd in the livers of some mice 2 days after dermal exposure to QD on intact skin^36^. Consistent with the findings in mice, another study found that QD could penetrate porcine skin^37^. The use of nanoparticles has also been explored in the colon where the imaging agent can be sprayed directly on a region of interest and excess reagent removed by irrigation^34, 38^.

Intravesical instillation is an established route of drug administration for bladder diseases. Intravesical immunotherapy with bacillus Calmette-Guerin (BCG) and chemotherapy with mitomycin are standard in bladder cancer management to reduce cancer recurrence and progression^39^. Adverse effects from intravesical administration of these agents are largely irritative symptoms to the lower urinary tract such as frequency and dysuria, although systemic absorption can occur, especially in the case of traumatic catheterization, and cause more serious adverse events including sepsis with BCG^40^. In our experiments, we tested a 100-fold higher concentration of anti-CD47-QD than what was previously used for imaging *ex vivo* human bladders (1 µM v. 10 nM)^10^. Using this increased concentration allowed us to track the biodistribution and assess toxicity of these nanoparticles at concentrations 1–2 orders of magnitudes higher than they might be used clinically. While all mice had detectable levels of QD in the bladder, systemic biodistribution of anti-CD47-QD in organs outside of the bladder was only observed in a subset of mice. Despite some systemic uptake and a high concentration of QD, blood test results did not indicate acute toxicity over a 7-day period. Inflammation was seen on histology in the renal pelvis of a subset of mice with QD exposure, but bladder tissue appeared normal following exposure to QD.

We speculate that the mechanisms of systemic uptake of anti-CD47-QD in some mice include direct penetration through the bladder wall, traumatic catheterization, and/or vesicoureteral (i.e. retrograde) reflux from the bladder to the kidney. The mice with exogenous Cd outside of the bladder fell into two groups: those that had the highest levels of Cd in the liver with correspondingly high levels in the kidneys and spleen, and those that had elevated levels of Cd primarily in the kidneys and bladder but not in other organs.

While the tight junction of the bladder urothelium limits the diffusion of intravesical agents into circulation, it is not completely impermeable, particularly in diseased states or trauma from catheterization. For example, in a randomized human study comparing efficacy and safety of mitomycin instillation, 1–5% of the instilled mitomycin was found systemically^41^. Compared to small molecule drugs like mitomycin (<500 Daltons), the QD used in our study is large (1.5–2 million Daltons and 15 nm diameter) and the *in vivo* fate of nanoparticles is related to their size, shape and surface chemistry^42^. While some accumulation of Cd in the kidney could be due to degradation of QD, this is unlikely. The CdSe core of the QD is encapsulated in a zinc sulfide polymer shell that is designed to prevent dissolution of free Cd. That there was no evidence of toxicity in our study or in the study of long term exposure QD exposure in rhesus macaques suggests^25^ that particle breakdown is quite slow. Thus, due to particle size, widespread systemic biodistribution is unlikely to be from penetration of an intact bladder wall, particularly since only a subset of mice had elevated Cd in multiple organs outside the bladder. We suspect that trauma due to catheterization created a vascular entry-point for the anti-CD47-QD and the damaged tissue led to systemic exposure. Further supporting this reasoning is the observation that most of the mice with systemic biodistribution were in Cohort 1. As we gained more experience catheterizing, the number of mice with significant Cd outside the urinary tract decreased, suggesting that there was a learning curve in mouse catheterization that have accounted for reduced systemic biodistribution in Cohort 2.

For mice that had elevated Cd confined to the urinary tract (i.e. kidney and bladder), we postulate the distribution pattern is related to vesicoureteral reflux of anti-CD47-QD from the bladder into the kidneys. While technically challenging, mouse bladder instillation is a well-established technique for testing intravesical therapies^43, 44^, for generation of orthotopic models of bladder cancer^45, 46^, and for treatment of bacterial infection^47^. To retain the intravesical agent and prevent efflux during incubation, a blocking strategy is usually employed. Strategies include tying the urethral opening^48^, clamping the catheter^43^ or connecting an air-filled syringe to the catheter^45^ as in our study. Mouse bladders only have approximately 150 µl capacity, in which we instilled 50 µl of solution. With a blocked catheter and potential urine production during incubation, it is possible that intravesical pressure increased resulting in reflux. Johnson *et al*. reported ~50% incidence rate of vesicoureteral reflux when instilling 50 µl of bacteria into mouse bladder^49^. Furthermore, systemic biodistribution to the organs outside of the urinary tract could be possible through pyelovenous backflow if intrarenal pressures are high enough. Additionally, even without significantly high pressures, the introduction of anti-CD47-QD to the kidney could lead to systemic exposure as the kidney is a well vascularized organ.

Despite noting some mice with systemic biodistribution, exposure to anti-CD47-QD did not translate into abnormalities in blood tests (Fig. 3) or changes in tissue histology (Fig. 4) within short term. IV administration of anti-CD47 caused transient anemia in mice with nadir at 5–7 days^50^, however this was not observed during the 7 days of this study with intravesical administration. BUN was slightly above the normal range on day 3 and 7 in QD treated mice and day 7 in anti-CD47-QD treated mice, but elevated BUN is a nonspecific finding which could indicate dehydration. Since the degree of BUN elevation was just above normal along with the nonspecific nature of the finding in a setting of otherwise normal blood test values, the finding is inconclusive particularly in the relatively short duration of this study.

On review of the histology, inflammation was observed in kidneys of a subset of QD-instilled mice. However, inflammatory cells were only found in the submucosal layer and the lumen of renal pelvis (Fig. 4b), suggesting inflammation was induced by vesicoureteral reflux. The thickness of urothelium is known to vary depending on bladder distention and location in the urinary tract of humans. For example, the urothelium is two to three cell layers thick in the minor renal calyces, three to five cell layers thick in the ureters, and six to seven cell layers thick in a contracted bladder. Upon bladder distention, however, the urothelium may flatten to two to three cell layers thick^51^. Thus, if mice have similar variation in urothelial thickness, the thinner urothelium in the renal pelvis may explain the inflammation found in the kidneys but not in the bladders of free CD47 instilled mice. Notably, the bladders of mice with inflammatory cells in the kidneys appeared normal with intact urothelium despite direct exposure to QD or anti-CD47-QD.

A limitation of our study is that we did not address QD biodistribution and toxicity in tumor-bearing mice. However, it is likely that the presence of bladder tumors would exacerbate systemic distribution after intravesical instillation. Additionally, as each animal was sacrificed for analysis at a certain time post instillation, our study lacks serial distribution data from the same mouse, which would be useful in assessing whether systemic distribution was due to initial trauma or vesicoureteral reflux or a slower migration of QD from the bladder. Further, the study provided only short term information for biodistribution and acute toxicity. The composition, size, shape or surface chemistry can all impact the *in vivo* distribution and toxicity of nanoparticles^24^. In this study only one size nanoparticle (15–20 nm) and one antibody conjugate were examined. Larger nanoparticles may be more likely to remain confined to the urinary tract and smaller nanoparticles may be more easily cleared through liver and kidney if they become systemic. Finally, the human urothelium is several cell layers thicker than mouse urothelium and the study of nanoparticle penetration of human bladder may reveal differences^52^.

In conclusion, intravesical administration of a nanoparticle labeled antibody remained largely confined to the urinary tract without evidence of systemic acute toxicity. Our study improves the understanding of the biodistribution pattern following intravesical instillation of nanoparticles. Due to the detection of QD outside of the urinary tract in some animals, further studies with longer term follow-ups are warranted, as well as investigation of other emerging nanoparticle-based imaging agents with favorable biosafety profile.

## Methods

### Anti-CD47-QD conjugation

Anti-CD47 (Rat anti-mouse CD47, clone miap301, 0.5 mg/ml) was conjugated to QD (Qdot_625_) using the SiteClick™ Qdot® 625 Antibody Labeling Kit (Invitrogen). QD was conjugated to the heavy chain by site-selective azide-DIBO cross-linking per manufacturer’s protocol and purified to remove unconjugated antibody.

### Bladder Instillation

All animal procedures were performed in accordance with protocols approved by the Stanford University Institutional Animal Care and Use Committee. Female C57BL/6 mice at 7–8 weeks age were catheterized transurethrally under anesthesia with a standard 24-gauge, 3/4 inch long angiocatheter^45^. Urine was removed through the catheter using a P200 pipette. Fifty microliter of PBS, QD (1 μM in PBS) or anti-CD47-QD (1 μM in PBS) was then pipetted into the hub of the catheter and instilled into the mouse bladder by gently depressing a 1 ml air-filled syringe attached to the catheter. After 30 min incubation, the syringe was aspirated to drain the bladder and the catheter was removed. Mice were returned to their cage after recovery from anesthesia or sacrificed for subsequent biodistribution and toxicity experiments at various time points.

### Intravesical imaging agent biodistribution using ICP-MS

For direct measurement of cadmium content of QD core, mice (n = 29) were sacrificed at 0, 1, 4, and 24 hrs following intravesical instillation of anti-CD47-QD to harvest blood (100 μl) and organs (heart, liver, spleen, lung, kidneys and bladder). For control (n = 3), blood and organs were harvested for comparison. Specimens were digested in 500 μl (blood and bladder), 750 μl (lung, spleen and heart), 1.5 ml (kidneys), or 4 ml (liver) of an acidic solution (67–70% HNO_3_ and 30–32% H_2_O_2_ 1:1) at 60 °C for 12 hrs. Samples were gently vortexed every hour to facilitate complete digestion. Tube caps were loosely closed to equilibrate pressure. After complete digestion, final volume in each tube was measured and 300 μl of each specimen was diluted to a final volume of 6 ml (20-fold dilution) with ultratrace analysis water. The diluted samples were filtered through 0.45 μm syringe filters before Cd determination using the ICP-MS (Thermo) at the Stanford Environmental Measurements Facility. Cd signal was measured per experimental parameters and instrument settings proposed by the manufacturer handbook. Standard solutions in ppb (μg/l) range (0.1–1000 ppb) were prepared from a commercial 1000 ± 4 mg/l Cd^2+^ stock solution. The limit of detection of ICP-MS for Cd was 0.009 ppb (µg/l), which was calculated as readout from blank solution plus 3 times standard deviation of 3 parallel readouts. Quality control standards (0.99 or 10.2 ppb) were measured every 20 specimens. The input anti-CD47-QD solution was diluted 100~1000-fold for measurement. The percent of instilled dose in a specific organ (% Instilled dose/organ) or 100 µl blood is calculated by the following equation:

1 $$\begin{array}{c} \% \,{\rm{instilled}}\,{\rm{dose}}/{\rm{organ}}\\ \quad =\frac{([QD]in\,tissue\,suspension)\times (volume\,of\,tissue\,suspension)\times (fold\,of\,dilution)\,}{([QD]in\,instilled\,solution)\times (volume\,of\,instilled\,solution)\times (fold\,of\,dilution)}\end{array}$$

### Toxicity Study

Two cohorts of mice were sacrificed at 1 day, 3 or 7 days post instillation of PBS, QD or anti-CD47-QD to harvest blood and organ specimens (heart, liver, spleen, lung, kidneys and bladder). As an additional control, the same specimens were collected from untreated mice. Blood specimens were submitted to the Animal Diagnostic Laboratory at Stanford University for tests including complete blood count, liver and kidney function tests. Organs were weighed and analyzed histologically by a clinical pathologist. Data from both cohorts were combined for analysis. Mice were excluded for analysis if signs of potential bladder perforation were observed, including blood during catheterization or in bladder drainage at the end of incubation.

### Statistical analysis

Quantitative variables were expressed using median and interquartile range, qualitative variables were expressed using size and percentage. For biodistribution study, ICP-MS results from Cohort 1 and 2 were combined for analysis. Data from biodistribution and toxicity were analyzed using analysis of variance (ANOVA) after verifying variance homogeneity and normal distribution of residuals. In case when the hypotheses were not verified a logarithmic transformation was performed. After all steps, if ANOVA hypotheses were still unverified, Kruskall-Wallis test was performed. In cases where the ANOVA test was statistically significant, *post*-*hoc* tests were performed and p values was adjusted using Tukey-Krammer method. The level of significance for each test was set to 5%. All statistical analysis in our study was carried out using SAS software version 9.4 (Cary, NC, USA), and graphics were generated using Prism 7 software (GraphPad, La Jolla, CA, USA).

## Electronic supplementary material

Supplementary Tables
