# Supplementary material for: In vivo biodistribution and toxicity of intravesical administration of quantum dots for optical molecular imaging of bladder cancer
Source: Sci Rep. 2017 Aug 24;7:9309. doi: 10.1038/s41598-017-08591-w (PMC5571179; doi:10.1038/s41598-017-08591-w)
Supplement: Supplementary file 1 — Supplementary Tables [file 41598_2017_8591_MOESM1_ESM.pdf]

**Supplementary files**

# In vivo biodistribution and toxicity of intravesical administration of quantum dots for optical molecular imaging of bladder cancer

Ying Pan[1,2,+], Timothy Chang[1,2,+], Gautier Marcq[1,2], Changhao Liu[3], Bernhard Kiss[1,2], Robert Rouse[2,4], Kathleen E. Mach[1,2], Zhen Cheng[3], Joseph C. Liao[1,2,*]

[1] Department of Urology, Stanford University School of Medicine, Stanford, CA 94305, USA

[2] Veterans Affairs Palo Alto Health Care System, Palo Alto, CA 94304, USA

[3] Department of Radiology and Molecular Imaging Program at Stanford, Stanford University School of Medicine, Stanford, CA 94305, USA

[4]Department of Pathology, Stanford University School of Medicine, Stanford, CA 94305, USA

[+] These authors contributed equally to this work

*Corresponding author: jliao@stanford.edu

**Supplementary Table S1. Cd amount (ng) in blood and organs of untreated and anti-CD47-QD-instilled mice in Figure 2**.

| | Untreated | | | Anti-CD47-QD-instilled | | | | | | | | | | | | | | | | | | | | | | | | | | | | |
|---|---|---|---|---|---|---|---|---|---|---|---|---|---|---|---|---|---|---|---|---|---|---|---|---|---|---|---|---|---|---|---|---|
| | | | | Cohort 1 | | | | | | | | | Cohort 2 | | | | | | | | | | | | | | | | | | | |
| | | | | 1 hr | | | 4 hrs | | | 24 hrs | | | 0 hr | | | | | 1 hr | | | | | 4 hrs | | | | | 24 hrs | | | | |
| **Mouse** | **1** | **2** | **3** | **1** | **2** | **3** | **1** | **2** | **3** | **1** | **2** | **3** | **1** | **2** | **3** | **4** | **5** | **1** | **2** | **3** | **4** | **5** | **1** | **2** | **3** | **4** | **5** | **1** | **2** | **3** | **4** | **5** |
| **Blood** | 0.3 | --* | 0.2 | 0.3 | 0.2 | 0.1 | 3.9 | 0.1 | 0.2 | 4.1 | 0.1 | -- | 0.2 | 0.2 | 0.2 | 0.1 | 0.2 | 0.1 | 0.2 | 0.1 | -- | 0.1 | -- | -- | 0.1 | 0.2 | -- | 0.2 | 0.1 | -- | -- | 0.2 |
| **Heart** | 0.2 | 0.1 | 0.2 | 0.2 | 0.3 | 0.6 | 0.8 | 0.3 | 0.3 | 4.5 | 0.2 | 0.2 | -- | 0.1 | -- | -- | 0.2 | 0.2 | -- | 0.2 | 0.1 | -- | -- | -- | 0.2 | -- | -- | -- | 0.2 | -- | 0.2 | -- |
| **Lung** | 0.2 | 0.3 | 0.2 | 0.5 | 0.2 | 1.1 | 1.7 | 0.9 | 0.5 | 4.0 | 0.3 | 0.3 | 0.2 | 0.2 | 0.3 | 0.2 | 0.2 | 0.9 | 0.4 | -- | 0.2 | 0.3 | -- | 0.2 | 0.2 | 0.2 | 0.2 | 0.2 | -- | 0.2 | 0.2 | 0.3 |
| **Spleen** | 0.6 | 0.1 | -- | 5.4 | 0.3 | 0.2 | 93.2 | -- | 0.4 | 187.5 | 0.4 | 0.2 | 0.2 | -- | 0.5 | 0.2 | -- | 0.5 | 0.2 | 0.2 | 0.3 | 0.2 | 0.3 | 0.2 | 0.2 | 0.2 | -- | 0.3 | 0.2 | 0.2 | 0.2 | 3.1 |
| **Kidneys** | 2.6 | 3.2 | 3.2 | 114.2 | 3.8 | 51.1 | 347.8 | 1.1 | 3.3 | 394.4 | 2.4 | 5.1 | 2.4 | 3.1 | 67.8 | 3.0 | 3.8 | 3.3 | 2.8 | 3.3 | 3.5 | 2.8 | 3.9 | 3.2 | 16.5 | 3.0 | 3.2 | 3.2 | 28.2 | 5.1 | 3.3 | 83.6 |
| **Liver** | 11.9 | 10.5 | 13.3 | 61.7 | 10.3 | 11.0 | 1507.1 | 8.9 | 9.9 | 3121.9 | 8.5 | 8.2 | 7.3 | 8.6 | 12.7 | 9.0 | 10.2 | 8.3 | 9.0 | 8.8 | 11.0 | 8.9 | 9.2 | 9.0 | 9.3 | 9.7 | 8.5 | 9.3 | 11.0 | 9.9 | 9.4 | 35.8 |
| **Bladder** | -- | 0.1 | 0.1 | 10.3 | 2.5 | 121.9 | 17.1 | 1.8 | 1.5 | 9.8 | 0.8 | 0.9 | 128.9 | 24.2 | 35.6 | 84.4 | 120.8 | 1.1 | 11.0 | 1.9 | 13.9 | 0.9 | 7.7 | 1.1 | 2.3 | 2.1 | 3.1 | 1.9 | 3.8 | 2.6 | 0.5 | 8.2 |

* undetectable

**Supplementary Table S2. Organ weight from untreated, PBS-, QD- and anti-CD47-QD-instilled mice in toxicity study.**

| | | Days post-instillation | No. of mice | Liver (mg) | | Spleen (mg) | | Kidney (mg) | | Lung (mg) | | Heart (mg) | | Bladder (mg) | |
|---|---|---|---|---|---|---|---|---|---|---|---|---|---|---|---|
| **Untreated** | | | 3 | 869* | 810-974** | 70 | 67-73 | 231 | 230-235 | 124 | 114-151 | 100 | 95-115 | 18 | 16-20 |
| **Treated** | **PBS** | 1 | 12 | 868 | 786-1022 | 77 | 71-89 | 249 | 224-267 | 129 | 123-138 | 105 | 101-119 | 20 | 17-22 |
| | | 3 | 12 | 836 | 786-900 | 75 | 65-91 | 239 | 221-256 | 130 | 123-137 | 113 | 95-123 | 15 | 14-19 |
| | | 7 | 12 | 856 | 745-951 | 74 | 68-88 | 245 | 230-251 | 136 | 125-142 | 106 | 103-113 | 16 | 13-21 |
| | **QD** | 1 | 6 | 822 | 745-852 | 70 | 69-89 | 235 | 230-245 | 134 | 125-152 | 92 | 88-117 | 17 | 16-19 |
| | | 3 | 7 | 826 | 758-855 | 96 | 83-112 | 255 | 237-270 | 137 | 123-142 | 100 | 97-148 | 20 | 17-23 |
| | | 7 | 6 | 853 | 815-993 | 76 | 73-81 | 260 | 238-264 | 141 | 129-143 | 112 | 98-123 | 22 | 16-24 |
| | **Anti-CD47-QD** | 1 | 6 | 981 | 869-1069 | 72 | 68-79 | 237 | 231-261 | 138 | 122-140 | 107 | 98-111 | 20 | 17-22 |
| | | 3 | 7 | 803 | 771-847 | 72 | 65-76 | 245 | 222-269 | 132 | 116-144 | 113 | 90-124 | 16 | 15-25 |
| | | 7 | 8 | 787 | 754-967 | 70 | 63-75 | 241 | 230-248 | 126 | 119-132 | 113 | 104-134 | 14 | 13-17 |

*Median with **interquartile range (IQR)
